# BEI Inactivated Vaccine Induces Innate and Adaptive Responses and Elicits Partial Protection upon Reassortant Betanodavirus Infection in Senegalese Sole

**DOI:** 10.3390/vaccines9050458

**Published:** 2021-05-04

**Authors:** Yulema Valero, José G. Olveira, Carmen López-Vázquez, Carlos P. Dopazo, Isabel Bandín

**Affiliations:** Departamento de Microbiología y Parasitología, Campus Vida, Instituto de Acuicultura, Universidade de Santiago de Compostela, 15782 Santiago de Compostela, Spain; jose.olveira@usc.es (J.G.O.); mdelcarmen.lopez.vazquez@usc.es (C.L.-V.); carlos.pereira@usc.es (C.P.D.); isabel.bandin@usc.es (I.B.)

**Keywords:** *Senegalese sole*, inactivated vaccine, BEI, formalin, nervous necrosis virus, immune response, antibodies, gene expression

## Abstract

Nervous necrosis virus (NNV), the causative agent of viral encephalopathy and retinopathy (VER), is one of the most threatening viruses affecting marine and freshwater fish species worldwide. Senegalese sole is a promising fish species in Mediterranean aquaculture but also highly susceptible to NNV and VER outbreaks, that puts its farming at risk. The development of vaccines for aquaculture is one of best tools to prevent viral spread and sudden outbreaks, and virus inactivation is the simplest and most cost-effective method available. In this work, we have designed two inactivated vaccines based on the use of formalin or binary ethylenimine (BEI) to inactivate a reassortant NNV strain. After vaccination, the BEI-inactivated vaccine triggered the production of specific IgM-NNV antibodies and stimulated innate and adaptive immune responses at transcriptional level (*rtp3*, *mx*, *mhcii* and *tcrb* coding genes). Moreover, it partially improved survival after an NNV in vivo challenge, reducing the mid-term viral load and avoiding the down-regulation of immune response post-challenge. On the other hand, the formalin-inactivated vaccine improved the survival of fish upon infection without inducing the production of IgM-NNV antibodies and only stimulating the expression of *herc4* and *mhcii* genes (in head-kidney and brain, respectively) during the vaccination period; this suggests that other immune-related pathways may be involved in the partial protection provoked. Although these vaccines against NNV showed encouraging results, further studies are needed to improve sole protection and to fully understand the underlying immune mechanism.

## 1. Introduction

Aquaculture is one of the fastest growing sectors of the food industry worldwide. Nowadays, global aquaculture has surpassed marine catches in terms of fish protein production for human consumption [1]. As a result of this growth, the spread of viruses has also risen, due to the constant transport of eggs and larvae between fish farms all over the world and because survivors of an epizootic outbreak often become asymptomatic carriers.

One of most widespread and dangerous viruses among fish farms is the nervous necrosis virus (NNV; Genus *Betanodavirus*, Family *Nodaviridae*). NNV is the causative agent of viral encephalopathy and retinopathy (VER), a neuropathological condition which provokes mortality rates of up to 100%, particularly at early stages of development (larvae and juveniles), especially in marine fish [2]. NNV is an icosahedral non-enveloped virus composed of two positive sense single stranded RNA segments—RNA 1 coding for the non-structural RNA-dependent RNA-polymerase, and RNA2 coding for the capsid protein. NNV is grouped into four different genotypes: red-spotted grouper nervous necrosis virus (RGNNV), barfin flounder nervous necrosis virus, tiger puffer nervous necrosis virus and striped jack nervous necrosis virus (SJNNV) [2]. In addition, natural reassortants between RGNNV and SJNNV genotypes have been isolated as the causative agents of disease outbreaks in different species, including sole (*Solea* sp.) and gilthead seabream (*Sparus aurata*), the latter previously thought to be an asymptomatic NNV carrier [3,4,5,6].

To date, the most common preventive strategies regarding husbandry and NNV-free broodstock selection appear to be inefficient at avoiding NNV outbreaks on fish farms due to the stability of the virus in the aquatic environment [2]. Therefore, vaccination stands as a cost-effective, innocuous and sustainable strategy to prevent NNV infections and severe episodes. Different strategies have been followed to design NNV vaccines, including live or inactivated viruses, DNA, virus-like particles, virus subunits, viral synthetic peptides or recombinant protein using diverse administration methods [7,8,9,10,11,12,13,14]. Although the immune response was triggered by most of them, they were only partially protective. As a first approach to inactivated dead recombinant bacteria, a spinycterin system expressing a downsized NNV coat protein was reported to induce 100% of sea bass (*Dicentrarchus labrax*) survival upon NNV infection [15]. However, the existence of contradictory data and the limited number of fish species on which vaccines have been tested make it difficult to release an effective and wide-spectrum vaccine against NNV onto the market. In fact, only two commercial formalin-inactivated vaccines against the RGNNV genotype, ALPHA JECT micro^®^ 1Noda (Pharmaq) and ICTHIOVAC^®^ VNN (Hipra), are available for sea bass vaccination in the Mediterranean area.

Senegalese sole (*Solea senegalensis*) is a highly promising flatfish species for aquaculture in Southern-European countries. However, one of the main drawbacks for its intensive farming is its high susceptibility to reassortant RGNNV/SJNNV (referring to the RNA1/RNA2 of the donors) strains [2]. In this work, a reassortant, sole pathogenic NNV strain has been used to design two inactivated vaccine formulations (using either formalin or binary ethylenimine, BEI). Juvenile sole individuals were intraperitoneally vaccinated and the antibody response and expression profiles of immune-related genes of vaccinated fish was assessed. In addition, fish survival upon NNV infection and the immunostimulation of challenged sole was also analyzed.

## 2. Materials and Methods

### 2.1. Viruses and Cells

The strain used in this study, SpSs-IAusc160.03 (hereafter Ss160), a reassortant strain exhibiting an RNA1 typed as RGNNV and an SJNNV-type RNA2, was isolated from Senegalese sole (*Solea senegalensis*) during an acute disease outbreak in a rearing facility in Spain in 2003 [3]. E-11 cell line was cultured in L-15 Leibovitz (Lonza) medium supplemented with 5% fetal bovine serum (FBS), penicillin (100 IU/mL) and streptomycin (100 mg/mL) at 25 °C. For viral propagation, cells were inoculated at an MOI = 0.1 and incubated in L-15 with 2% FBS at 25 °C until the cytopathic effect (CPE) was extensive. The supernatant was harvested and centrifuged to eliminate cell debris. Virus stock was titrated in 96-well plates and expressed as the viral dilution infecting 50% of the cell cultures (TCID_50_), following a previously defined methodology [16].

### 2.2. Vaccine Inactivation

Either formalin or binary ethylenimine (BEI) was used to inactivate Ss160 (iSs160; 10^8.5^ TCID_50_/mL). Formalin inactivation was accomplished by adding the reagent into the viral suspension to a final concentration of 0.2% during 7 or 9 days (d) at 25 °C in agitation. BEI inactivation was achieved by mixing the virus with freshly prepared 0.1 M BEI dissolved in 0.175 M NaOH to a final concentration of 0.1, 1 or 4 mM. Inactivation was tested after 1, 24, 48 or 72 h of inactivation at 25 °C in agitation. After incubation, 1 M sodium thiosulphate was added (1:10 in relation to BEI). Vaccines were confirmed to be completely inactivated by the absence of CPE and viral titer in the E-11 cell line, after three 10-day blind passages, as elsewhere [3]. In addition, in vivo toxicity was ruled out by intraperitoneal injection (ip) in sole juveniles.

### 2.3. Animals and Sampling Procedure

A total of 920 healthy Senegalese sole juveniles (2.9 ± 0.1 g body weight) from a local farm were transferred to the *Universidade de Santiago de Compostela* (Spain) aquarium facilities. Fish were randomly divided into 300 L running seawater tanks (33‰ salinity) at 19 °C and with a 12 h light: 12 h dark photoperiod and acclimatized for 15 days prior to the experiments. During acclimatization, fish were tested for the presence of NNV by RT-qPCR (see below). Twenty fish were reserved to be mock-vaccinated and -infected until the end of the trials.

Sampled fish were analyzed as follows: blood was collected from the caudal peduncles and serum samples were obtained by centrifugation at 10,000× *g* for 10 min at 4 °C and immediately stored at −20 °C until use. Afterwards fish were sacrificed with a tricaine methanesulfonate overdose (MS-222, Sigma-Aldrich, St. Louis, MO, USA). Head-kidney and brain were removed and immediately stored at −80 °C until later use for RT-qPCR assays and cell culture when applicable.

### 2.4. Fish Vaccination

Senegalese sole specimens were randomly divided into ten 100 L tanks (n = 90/tank), forming five experimental groups in duplicate. Fish were gently sedated with MS-222 and intraperitoneally (ip) vaccinated as follows: Control group (Control) was injected with PBS (100 μL/fish) while vaccinated groups received a single ip injection of Ss160 inactivated with either formalin (form-iSs160) or BEI (BEI-iSs160) with high and low dose, 10^5^ (iSs160L) and 10^7^ TCID_50_/mL (iSs160H), respectively. After vaccination, fish (n = 6 fish/group and time point) were sampled at 7-, 15-, and 30-days post-vaccination (dpv) as described above.

### 2.5. NNV Challenge

Thirty days after vaccination, the remaining fish were challenged by immersion and exposed to an Ss160 concentration of 10^5^ TCID_50_/mL for 3 h with strong aeration (dissolved oxygen concentration 11 ± 1 mg/L) at 22 °C. Control fish were mock-infected with L-15 medium. Mortalities and clinical signs were recorded daily. Viral load quantification in brain tissue was performed on fish (n = 3 fish/group) sampled at 12-day intervals from the initial detection of VER signs (see below). After 45 days of infection, serum, head-kidney and brain samples from surviving fish (n = 6 fish/group) were analyzed.

### 2.6. Specific and Neutralizing Antibody Levels

The detection of specific antibodies against NNV (IgM-NNV) was performed by a previously described indirect ELISA with slight modifications [17]. Briefly, 20 μg of total proteins from serum samples were diluted in coating buffer [100 mM Bicarbonate/Carbonate, pH 9.6] and incubated overnight at 4 °C in 96 High Binding flat-bottomed plates (Sarsted, Newton, NC, USA). After washing with PBST [PBS with 0.2% of Tween-20], the samples were blocked with 5% skimmed milk in PBST for 1 h. Afterwards, incubation with a rabbit anti-NNV (Abcam, Inc., Hong Kong; 1:10,000) was performed for 1 h at room temperature. After three 5-min washes, the samples were incubated with the anti-rabbit IgG-HRP (Sigma Aldrich; 1:25,000) for 1 h at room temperature. The reaction was revealed with 100 μL per well of 3,3′,5,5′-tetramethylbenzidine single solution (ThermoFisher, Waltham, MA, USA) for 20 min and stopped with 50 μL of 2 M sulphuric acid. The absorbance was read at 450 nm with an iMark™ Microplate Absorbance Reader (BioRad, Heracles, CA, USA). All assays were performed in duplicate. Previously assayed positive serum was used as a positive control whilst the absence of sample, NNV, primary or secondary antibodies were used as negative controls.

Neutralizing antibodies were assayed as elsewhere [14], with few modifications. In brief, sera from fish obtained during vaccination and after 45 days post-challenge (dpc) were decomplemented at 56 °C during 30 min. Serial dilutions (from 10 to 640-fold) of decomplemented sera were incubated with equal volumes of 10^2.5^ TCID_50_ mL of Ss160 for 1 h at 25 °C. After incubation, samples were assayed for NNV replication on E-11 cells as above, and serum dilution to provoke the absence of CPE was determined. A serum from an infected fish served as a positive control.

### 2.7. Gene Expression by Real-Time Polymerase Chain Reaction

Total RNA was isolated from head-kidney and brain using an EZNA Total RNA purification Kit (VWR) following the manufacturer’s instructions. The RNA samples were resuspended in 70 µL of nuclease-free water (VWR), quantified by absorbance at 260 nm in a Nanodrop ND-100 spectrophotometer (Nanodrop Technologies) and stored at −80 °C. The first strand of cDNA was synthetized using Superscript IV (ThermoFisher) with Random Hexameres (ThermoFisher) as previously described [18].

The expression of sole immune-related genes, namely the receptor transporter protein (*rtp3*), E3 ubiquitin-protein ligase (*herc4*), interferon-induced GTP-binding protein Mx (*mx*), major histocompatibility complex II (*mhcii*) and T-cell receptor beta (*tcrb*), were analyzed by RT-qPCR, in an iCycler iQ CFX96TM Real Time System (BioRad) following the manufacturer’s instructions using iQTM SYBR^®^ Green Supermix (BioRad). Reaction mixtures (containing 20 μL of SYBR Green supermix with 0.2 μM of the specific primers and 2 μL of cDNA template) were incubated for 3 min at 95 °C as an activation/denaturation step, followed by 40 cycles of 15 s at 95 °C and 30 s at 55 (*mhcii*, *mx* and *rtp3*) or 58 °C (*tcrb* and *herc4*). The specific primers used are shown in Table 1. Negative controls with no template were always included in the reactions. The relative expression of all genes was calculated by the 2^−ΔΔCt^ method [19] using the beta actin (*actb*) coding gene as the endogenous reference.

### 2.8. Viral Quantification

Betanodavirus RNA1 extraction and amplification was accomplished as described above using SnodR1 primers (Table 1). The corresponding standard curve was prepared using 20-fold dilutions of a plasmid containing the full-length RNA1 of strain Ss160 in the range of 10^1^ to 10^7^ copies/μL. Viral load data were calculated as RNA1 copies per g of fish tissue. All samples were tested in triplicate.

### 2.9. Calculations and Statistics

All the data are shown as the mean ± standard error of the mean (SEM). The data corresponding to the mRNA transcriptional levels was expressed as the relative gene expression of the control or vaccinated group. IgM-NNV results are described as the optical density (OD) at 450 nanometers. The relative percent survival (RPS) was calculated from the cumulative mortality by the following formula:

(1) $$RPS=\left[\left(1-\frac{\%\;mortality\;of\;vaccinated\;group}{\%\;mortality\;of\;control\;group}\right)\;*\;100\right].$$

Variations between different time points and groups were analyzed by a two-way ANOVA followed by Tukey’s post-hoc analysis. A non-parametric Kruskal-Wallis test, followed by a multiple comparison test, was used when data did not meet parametric assumptions. Survival rates were compared between groups using the Kaplan Meier test. Letters denote statistical differences between groups at a same time-point (*p* < 0.05), whilst asterisks indicate those differences between time-points in the same group (* *p* < 0.05; ** *p* < 0.01; **** *p* < 0.0001). All statistical analyses were conducted using GraphPad Prism 8.

## 3. Results

Prior to the vaccination assays, fish were tested by RT-qPCR and were confirmed to be free of NNV (data not shown). In addition, five fish per vaccine formulation were ip injected and monitored for 48 h before the beginning of the vaccination experiments to rule out the toxicity of the inactivated vaccines. A control group with mock-vaccinated and infected specimens was maintained until the end of the experiments as tank-effect control.

### 3.1. Formalin and BEI Effectively Inactivated Ss160

The effectivity of inactivation was tested using different chemical concentrations and incubation times (Table 2). Complete formalin inactivation was achieved after nine days of incubation since neither CPE nor viral titer were recorded after three blind passages (Table 2). In the case of BEI, inactivation was accomplished with 1 mM after 72 or 4 mM at any time point tested (Table 2). Based on these results, a form-iSs160 vaccine (0.2% formalin for nine days) and a BEI-iSs160 vaccine (1 mM BEI for 72 h) were chosen for in vivo vaccination assays. After 7, 15 and 30 dpv, NNV was not recovered in cell culture from the brain of vaccinated sole (three brains individually, data not shown) corroborating the complete inactivation of the vaccines chosen.

### 3.2. BEI-iSs160 Vaccine Induces the Production of Specific NNV-IgM

**Figure 1:**
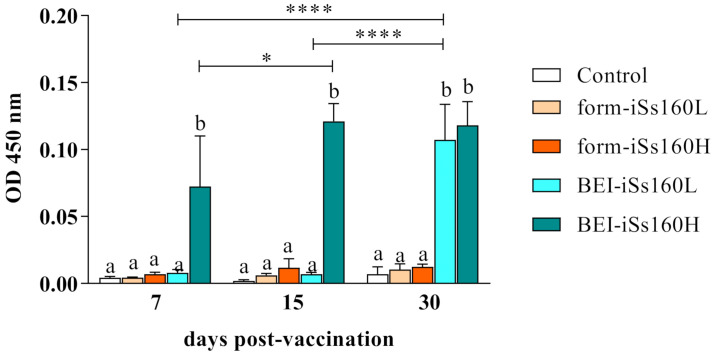
BEI-iSs160 vaccine induces specific humoral immunity. Specific anti-NNV IgM levels in the serum of Senegalese sole specimens 7-, 15- and 30-days after intraperitoneal vaccination with low and high dosages (10^5^ and 10^7^ TCID_50_/mL, iSs160L and iSs160H, respectively) of BEI-iSs160 vaccine (inactivated with BEI) and form-iSs160 (formalin inactivated) or PBS (Control). Data represent the mean ± standard error of the mean (SEM; n = 6 fish/group and time point). Lower letters denote statistical differences between groups at a same time-point (*p* < 0.05), whilst asterisks indicate those between time-points in the same group (* *p* < 0.05; **** *p* < 0.0001).

After 7, 15 and 30 dpv, the production of specific antibodies against NNV was studied (Figure 1). Only the BEI-iSs160 vaccine induced the synthesis of NNV-IgM from 7 dpv onwards or after 30 dpv (iSs160H or iSs160L, respectively). Moreover, this increase was significant with time in both BEI-iSs160 vaccinated groups, although no neutralizing activity was observed (data not shown). On the contrary, form-iSs160 vaccines did not provoke the production of specific antibodies at any time tested.

### 3.3. Immune-Related Gene Transcription in Vaccinated Fish

**Figure 2:**
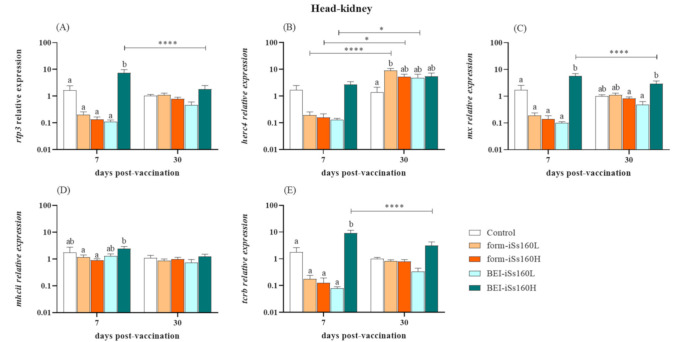
Vaccination produces the increment of immune response at transcriptional level in head-kidney. Expression of immune-related genes (**A**) *rtp3*, (**B**) *herc4*, (**C**) *mx*, (**D**) *mhcii* and (**E**) *tcrb* in the head-kidney of *Senegalese sole* specimens 7-, 15- and 3-days after intraperitoneal vaccination with low and high dosages (10^5^ and 10^7^ TCID_50_/mL, iSs160L and iSs160H, respectively) of BEI-iSs160 vaccine (inactivated with BEI) and form-iSs160 (formalin inactivated) or PBS (Control). Data represent the mean ± standard error of the mean (SEM; n = 6 fish/group and time). Lower letters denote statistical differences between groups at a same time-point (*p* < 0.05), whilst asterisks indicate those between time-points in the same group (* *p* < 0.05; **** *p* < 0.0001).

**Figure 3:**
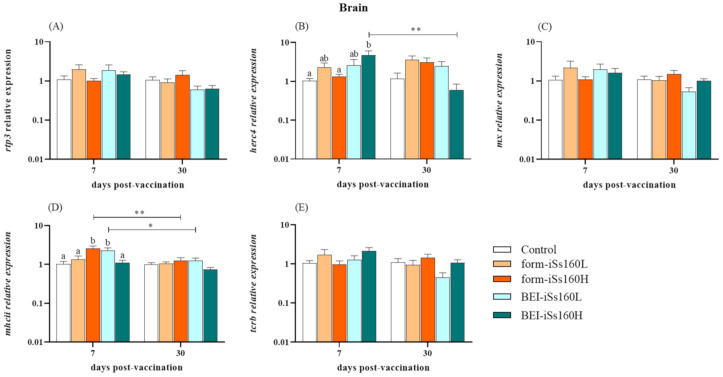
Vaccination barely alters the immune response at transcriptional level in brain. Expression of immune-related genes (**A**) *rtp3*, (**B**) *herc4*, (**C**) *mx*, (**D**) *mhcii* and (**E**) *tcrb* in the brain of *Senegalese sole* specimens 7-, 15- and 30-days after intraperitoneal vaccination with low and high dosages (10^5^ and10^7^ TCID_50_/mL, iSs160L and iSs160H, respectively) of BEI-iSs160 vaccine (inactivated with BEI) and form-iSs160 (formalin inactivated) or PBS (Control). Data represent the mean ± standard error of the mean (SEM; n = 6 fish/group and time). Lower letters denote statistical differences between groups at a same time-point (*p* < 0.05), whilst asterisks indicate those between time-points in the same group (* *p* < 0.05; ** *p* < 0.01).

At the gene expression level, most of the studied immune-related markers were modulated in the head-kidney of vaccinated fish, whilst in the brain they were barely altered (Figure 2 and Figure 3). Thus, in head-kidney, an overexpression of *rpt3*, *mx* and *tcrb* genes was observed in the BEI-iSs160H group compared to the controls and the other vaccinated groups after 7 dpv. However, a significant decrease down to control levels was observed after 30 dpv in the transcription of the three genes (Figure 2A,C,E). Regarding the fish vaccinated with form-iSs160, only the form-iSs160L triggered increased transcriptional levels of *herc4* at 30 dpv (Figure 2B). Finally, although *mhcii* gene expression was unaltered in vaccinated groups compared to the controls, at 7 dpv in the BEI-iSs160H group, up-regulated levels were observed when compared with formalin vaccinated fish (Figure 2D). In brain tissue, only a few changes were seen at the short time point (7 dpv; Figure 3). Thus, *herc4* gene expression was increased in the BEI-iSs160H group whilst *mhcii* was increased in the form-iSs160H and BEI-iSs160L groups (Figure 3D). In all cases, gene expression returned to control levels after 30 dpv (Figure 3B,D).

### 3.4. BEI-iSs160 Vaccine Improves Survival and Reduces Viral Load in Sole Brain after Challenge

**Figure 4:**
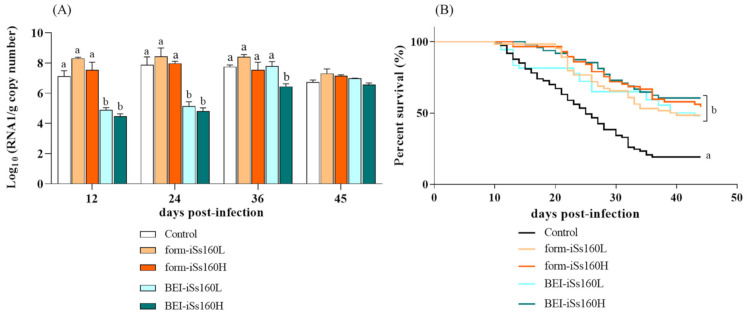
All vaccines induce partial protection and BEI-iSs160 decreases the viral load after challenge. (**A**) viral load (n = 3 fish/group and time) in brain and (**B**) Percent of survival during 45 days of in vivo infection in intraperitoneally vaccinated *Senegalese sole* specimens vaccinated with low and high dosages (10^5^ and 10^7^ TCID_50_/mL, iSs160L and iSs160H, respectively) of BEI-iSs160 vaccine (inactivated with BEI) and form-iSs160 (formalin inactivated) or PBS (Control). Infection was performed by 3 h of immersion with 10^7^ TCID_50_/mL. Survival rates were compared between groups using Kaplan Meier test. Lower letters denote statistical differences between groups of the same time point according to the two-way ANOVA test (*p* < 0.05).

At 30 dpv, fish were bath-challenged with the Ss160 strain and at 12 dpc, control fish started to show typical symptoms of VER disease such as erratic swimming or changes in skin color (data not shown), whereas no external disease signs were observed in the vaccinated fish. Therefore, from this time point and at 12-day intervals the viral load in the fish brain tissue (n = 3 fish/group and time point) was individually assessed. From 12–24 dpc, in the BEI-vaccinated groups the viral load was significantly lower (8.82 × 10^4^–1.98 × 10^5^ and 3.46 × 10^4^–7.99 × 10^4^ RNA copies per gram in the BEI-iSs160L and BEI-iSs160H groups, respectively) than in the control fish (2.71 × 10^8^ and 7.91 × 10^7^ copies) (Figure 4A). However, at 36 dpc viral RNA copies only remained low in the BEI-iSs160H group (3.3 × 10^6^ *versus* 6.2 × 10^7^ in the control) and at the end of the challenge (45 dpc) no significant differences were found between vaccinated and control fish. No variations with the control group were observed in both form-iSs160 vaccinated groups at the time-point assayed (Figure 4A).

After 45 dpc, a significant improvement of survival was observed with all vaccines tested (Figure 4B), although the best results were obtained with the high concentrations. Thus, whereas in the control group only 19.2% of the individuals survived, form- and BEI-iSs160H groups displayed survival values of 54.38 and 60.42%, respectively (RPS 43.7 and 51.0). Low vaccine concentrations showed very similar RPS values (35.8 and 36.2, BEI- and form-iSs160L, respectively).

### 3.5. NNV Infection Induces the Production of Specific NNV-IgM Similarly in All Fish

**Figure 5:**
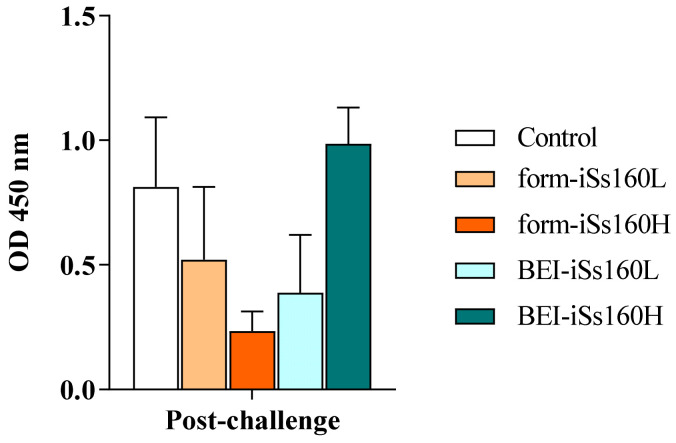
Vaccination does not alter specific humoral immunity upon NNV infection. Specific anti-NNV IgM levels in the serum of *Senegalese sole* specimens 45 days after 3 h of immersion with 10^5^ TCID_50_/mL of Ss160 in previously intraperitoneally vaccinated *Senegalese sole* specimens vaccinated with low and high dosages (10^5^ and 10^7^ TCID_50_/mL, iSs160L and iSs160H, respectively) of BEI-iSs160 vaccine (inactivated with BEI) and form-iSs160 (formalin inactivated) or PBS (Control). Infection was performed by 3 h of immersion with 10^7^ TCID_50_/mL. Data represent the mean ± standard error of the mean (SEM; n = 6 fish/group and time). Statistical assay was performed by the two-way ANOVA test (*p* < 0.05).

The level of specific NNV-IgM was analyzed 45 dpc (Figure 5). The in vivo infection elicited the production of specific antibodies in vaccinated and control fish with no statistical differences between groups. In addition, no NNV-neutralizing activity was recorded (data not shown).

### 3.6. Immune-Related Genes in Head-Kidney after NNV Challenge

**Figure 6:**
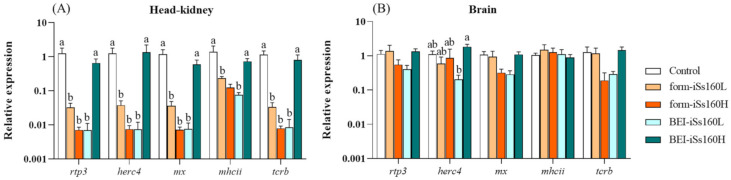
Vaccination mainly decreases the immune response upon NNV infection at a transcriptional level in head-kidney but not in brain. Expression of immune-related genes in (**A**) head-kidney and (**B**) brain of *Senegalese sole* specimens 45 days after 3 h of immersion with 10^5^ TCID_50_/mL of Ss160 in fish previously vaccinated with PBS (Control) or with low and high dosages (10^5^ and 10^7^ TCID_50_/mL, iSs160L and iSs160H, respectively) of BEI-iSs160 vaccine (inactivated with BEI) and form-iSs160 (formalin inactivated) or PBS (Control). Data represent the mean ± standard error of the mean (SEM; n = 6 fish/group and time). Lower letters denote statistical differences between groups according to the two-way ANOVA test (*p* < 0.05).

At 45 dpc, the analyzed genes were down-regulated in the head-kidney of all fish treated with the formalin-inactivated vaccine. This down-regulation was also observed in the BEI-iSs160L group, whereas it remained unaltered in the BEI-iSs160H vaccinated fish (Figure 6A). In the brain, the mRNA transcriptomic levels of the studied immune-related genes were unaltered in all vaccinated fish when compared with the control group (Figure 6B). However, comparison among vaccinated groups showed that *herc4* gene expression was significantly higher in the BEI-iSs160H than in the BEI-iSs160L group (Figure 6B).

## 4. Discussion

Although *Senegalese sole* is a promising species for Mediterranean aquaculture that shows extraordinary susceptibility to NNV [18,21], there is only one study describing an experimental recombinant NNV vaccine for this fish species which induced a good antibody response but whose protective effect was not assessed [22]. Therefore, aiming to prevent VER disease in sole, and considering that the simplicity of the production method makes the inactivated vaccines one of the most practical, we have designed a BEI- and a formalin-inactivated vaccine using the sole-pathogenic reassortant strain, Ss160.

The main concept linked to the inactivation of viruses for vaccine production is to generate totally safe vaccines, with the complete blockage of viral replication, without damaging its antigenicity [23]. Our first observation was that the complete inactivation of NNV was achieved with 0.2% formalin or 1 mM BEI after 9 days or 72 h of incubation, respectively. Previously, NNV has shown a certain resistance to be inactivated by low concentrations of formalin (up to 0.16%), needing concentrations of at least 0.5% or higher [24,25,26,27]. In the case of BEI, a previous concentration used to block NNV replication was 4 mM [7,26,28,29]. Consumers today tend to be increasingly concerned about the origin and the composition of the food they eat, so it may be worth reducing any chemical compounds used in vaccination strategies, as done in this work, to avoid a generalized rejection of the product but ensuring, at the same time, that NNV is completely inactivated with no possibility of being spread into the wild.

Our post-vaccination results showed the abrupt induction of specific IgM-NNV production in BEI-iSs160 vaccinated fish, in a concentration dependent manner, although they were not neutralizing. Similarly, other BEI inactivated NNV vaccines for grouper strains triggered the synthesis of IgM-NNV, but they also possessed neutralizing activity [28,29]. On the contrary, fish immunized with the form-iSs160 vaccine did not elicit an antibody response. Most of the formalin inactivated vaccines against NNV reported in the literature reveal a significant increment in neutralizing antibodies, regardless of whether the sera are decomplemented or not [26,27,30,31,32,33,34,35,36]. However, formalin-inactivated vaccines appear to be dose dependent, since dosages of around 10^7^ TCID_50_/fish are necessary to provoke an effective production of specific NNV antibodies in several fish species [27,31,32,33,34,35,36]. As a matter of fact, the minimum vaccine concentration to successfully generate neutralizing antibodies in groupers is 10^7^ TCID_50_/fish [36]. Therefore, it is reasonable to assume that the lack of antibody production observed in sole is due to the low vaccine concentration used in this study (10^6^ and 10^4^ TCID_50_/fish).

To the best of our knowledge, this is the first study addressing the immune response at a transcriptional level of sole immunized with NNV inactivated vaccines. Interestingly, the transcription of *rtp3*, *mx*, *herc4* and *tcrb* (all of them demonstrated to be up-regulated in NNV-infected *S. sole* [20]) was considerably induced in the head-kidney of fish vaccinated with the BEI-iSs160H, whereas in brain tissue it was barely altered. Thus, the gene expression of *rtp3*, a virus-responsive gene [37], was increased in the kidney soon after vaccination (7 dpv) although the values were similar to the control fish at 30 dpv. This gene was also greatly up-regulated in the head-kidney and brain of sole infected with the reassortant Ss160 strain [20], just like in the brain and in several organs of NNV-infected Atlantic salmon (*Salmo salar*) and Asian seabass (*Lates calcarifer*), respectively [37,38]. The *rtp3* gene shows an extraordinary sensitivity to NNV since its up-regulation is highly stimulated soon after infection, appearing to be linked to the resistance to infection [38,39]. This information, taken together with our results, points to Rtp3 as a relevant protein in the fight against NNV. However, further studies are needed to clarify its specific function. In a similar manner, the *mx* gene expression peaked after 7 dpv, perfectly simulating a natural NNV infection. As a matter of fact, *mx* gene stimulation has usually been used as an indicator of the vaccine’s antiviral effectiveness due to the significant induction of the type I interferon (IFN) route—the most powerful antiviral pathway—in fish infected with NNV [20,40,41,42,43,44]. This induction seems to be regardless of the administration method, given that other tested BEI-vaccines showed a suitable *mx* gene up-regulation during the vaccination period, when administered either by bath or intraperitoneal injection [7,28]. Finally, the BEI-vaccine seems to organize complete adaptive cellular immunity in head-kidney because *tcrb* (a marker of cytotoxic T cells subsets) and *mhcii* (marker of T helper cells route activation via antigen presenting cells) genes were up-regulated 7 days after vaccination, as seen in groupers [7,28], concomitantly with the induction of specific IgM-NNV production observed. It has previously been described how T cells were activated upon in vivo infection with NNV [41,45,46,47] and even after vaccination with UV-inactivated, recombinant or DNA vaccines [8,14,48], as with the BEI-iSs160H vaccine. Exceptionally, the *herc4* gene was the only gene up-regulated in the brain after BEI vaccination but not in head-kidney. Although its function in fish is still unclear, the *herc4* gene is up-regulated in the head-kidney and brain of infected susceptible fish whilst inhibited in asymptomatic carriers of NNV [20,49]. In addition, this molecule seems to be relevant in antiviral responses, as the viral mimic poly (I:C) in macrophages induced an increment of its transcriptional levels [50]. Regarding the formalin-inactivated vaccines, only *herc4* was overexpressed in the head-kidney of fish injected with the form-iSs160L, whereas *mhcii* transcriptional levels were increased in the brain of fish vaccinated with the form-iSs160H. Several formalin inactivated vaccines have been reported to confer protection against NNV in different fish species [26,27,31,32,33,34,51], but immune-related gene expression was only studied in European sea bass [30]. In agreement with our results, *mx* was not significantly overexpressed in kidneys although a significant increase was observed in the gut at 48 h post-vaccination [31].

Thirty days after vaccination, fish were bath-challenged. Although mortalities appeared approximately at the same time as in most of the groups, only control fish showed VER disease signs, such as erratic swimming and alterations in the skin color. The absence of symptomatology coincided with the improvement in the survival of vaccinated fish, especially those injected with BEI-iSs160H, which showed an RPS value of 51, whereas survival of control fish was only 19.2%. Our results agree with those found in groupers of different ages, where BEI-inactivated NNV triggered partial survival rates ranging between 52–67% in early juveniles when ip challenged [28]. However, no differences were shown in the production of specific antibodies at the end of the challenge, contrary to what happens in groupers immunized either with formalin- or BEI-inactivated vaccines [27,28,32,36]. In fact, in this fish species, a potent humoral response against NNV has been associated with the improvement in the survival of vaccinated fish [32]. Our results indicate, therefore, that cellular immunity can play an important role in the partial protection achieved in sole, and that the form-iSs160 vaccine could induce additional defense mechanisms to those analyzed in this study. Regarding survival rates provoked by formalin-inactivated vaccines against NNV, there are many controversial results in which survival rates range from 10.1–100% with an unclear common pattern [26,27,31,32,35,36]. Many variables may be affecting the differential effectivity of this type of vaccine, such as the viral strain used, the formalin dosage and inactivation period, the vaccine dosage or the virus load used in the challenge. However, the vaccine dosage appears to be the most plausible explanation as it is determinant for triggering neutralizing antibodies. Nevertheless, further studies might be necessary to fully understand the conditioning factors for the effectivity of this type of vaccine.

After the challenge, we observed a generalized down-regulation of all the genes studied in the head-kidney of fish vaccinated with form-iSs160 (both concentrations). However, the BEI-iSs160H was the only one maintaining transcriptional levels of immune-related genes with no inhibition. This may be relevant given that NNV has been reported to eradicate the innate immune response during the first moments of infection to freely spread and colonize target tissues [40,52]. Finally, and despite the similarities in the survival rates found in all groups, it is worth noting that BEI-iSs160H, in addition to maintaining the transcriptional levels of immune-related genes, provoked a significant decrease in the viral load in the brain tissue of challenged fish from 12–36 dpc, coinciding with the onset of disease signs (day 12 post challenge) and the period of higher mortalities in control fish (20–30 dpc).

## 5. Conclusions

NNV inactivated vaccines generated in this study conferred partial protection to *Senegalese sole* when administered by ip injection, although they induced a different immune response. On the one hand, BEI-iSs160 generates the innate (type I interferon pathway, *rtp3*) and adaptive (specific IgM-NNV production, T cell markers) immune responses which last for at least 30 days. This vaccine improves sole survival upon NNV infection by decreasing the viral load in brain tissue and maintaining their immune response unaltered until the end of the challenge. The consistency of the results obtained with BEI inactivation ([28] and this study) suggest that the reassortant strain Ss160 inactivated with BEI has the potential to be used for developing vaccines which generate a robust immune response in *S. sole*. However, further experimental approaches are needed to improve this promising vaccine, including either booster immunizations or adjuvants. On the other hand, form-iSs160 decreases the mortality rates upon NNV infection even when a scarce immune stimulation was observed, pointing to the fact that different immune-related pathways are involved.

## Figures and Tables

**Table 1:** Primer sequences used for gene expression analysis.

	Protein Name	Gene Name	Accession Number, UniGen Name or Reference	Sequence (5′–3′)	
Nodavirus	RNA-dependent RNA polymerase	RNA1	FJ803911	F	TCCAAAAGAAAGAAGCATAC
				R	TGGCATGTACCACGGAAC
Senegalese sole	Receptor transporter protein	*rtp3*	[20]	F	GACGCCCCAATGGTGGAT
				R	CCAGATTCTTCATGAGGATGGTGAT
	E3 ubiquitin-protein ligase	*herc4*	[20]	F	GCCAAAACACTGGCATGGTT
				R	AACGCCAAACAGGAAGTACCT
	Interferon-induced GTP-binding protein Mx	*mx*	[20]	F	CCTCTCTCCTTCAGGATCCTCCTCCTGTGC
				R	CAAAACAAGAAACTATCTGCCTGGTGGTTC
	Major histocompatibility complex II	*mhcii*	[20]	F	CGCTGATGAAAATGATCCACCTTCT
				R	ACCAGTCACATGACAGATCAGAGT
	T-cell receptor beta chain	*tcrb*	solea_v4.1_unigene681812 *	F	CAGGAGGCACAGCTATGAAA
				R	TCTCCACCCAAATCTCCAAA
	Beta actin	*actb*	DQ485686	F	GACGACATGGAGAAGATC
				R	GGTGTTGAAGGTCTCAAA

* NOTE: UniGen transcriptomic database in http://www.scbi.uma.es/soleadb (accessed on 21 February 2021).

**Table 2:** Safety test of form- and BEI-iSs160 vaccines after different concentration of chemical compounds treatments and incubation periods.

Chemical Compound	Concentration		Time		CPE under Each Blind Passage			Titer after Virus Inactivation(Log_10_ TCID_50_/mL)
	%	mM	Days	Hours	1	2	3	
Formalin	0.2		7		−	−	+	nt
			9		−	−	−	<1.0
BEI		0.1		1	+	nt	nt	nt
				24	+	nt	nt	nt
				48	+	nt	nt	nt
				72	+	nt	nt	nt
		1		1	+	+	nt	nt
				24	+	+	nt	nt
				48	−	−	+	nt
				72	−	−	−	<1.0
		4		1	−	−	−	<1.0
				24	−	−	−	<1.0
				48	−	−	−	<1.0
				72	−	−	−	<1.0

NOTE: −: negative; +: positive; nt: non-tested.

## Data Availability

Not applicable.
